# The nature of chronic rejection after lung transplantation: a murine orthotopic lung transplant study

**DOI:** 10.3389/fimmu.2024.1369536

**Published:** 2024-04-25

**Authors:** Tobias Heigl, Janne Kaes, Celine Aelbrecht, Jef Serré, Yoshito Yamada, Vincent Geudens, Anke Van Herck, Arno Vanstapel, Annelore Sacreas, Sofie Ordies, Anna Frick, Berta Saez Gimenez, Jan Van Slambrouck, Hanne Beeckmans, Nilüfer A. Acet Oztürk, Michaela Orlitova, Annemie Vaneylen, Sandra Claes, Dominique Schols, Greetje Vande Velde, Jonas Schupp, Naftali Kaminski, Markus Boesch, Hannelie Korf, Schalk van der Merwe, Lieven Dupont, Jeroen Vanoirbeek, Laurent Godinas, Dirk E. Van Raemdonck, Wim Janssens, Ghislaine Gayan-Ramirez, Laurens J. Ceulemans, John E. McDonough, Erik K. Verbeken, Robin Vos, Bart M. Vanaudenaerde

**Affiliations:** ^1^ Laboratory of Respiratory Diseases and Thoracic Surgery, KULeuven and UZ Gasthuisberg, Leuven, Belgium; ^2^ Department of Thoracic Surgery, Kyoto University Hospital, Kyoto, Japan; ^3^ Translational Cell and Tissue Research, KU Leuven and UZ Gasthuisberg, Leuven, Belgium; ^4^ Pulmonology Service, Lung Transplant Program, Hospital Universitari Vall d’Hebrón, Barcelona, Spain; ^5^ Department of Respiratory Medicine, Uludag University Faculty of Medicine, Bursa, Türkiye; ^6^ Department of Imaging and Pathology, Biomedical MRI/MoSAIC, KU Leuven, Leuven, Belgium; ^7^ Pulmonary, Critical Care and Sleep Medicine, Yale University School of Medicine, New Haven, CT, United States; ^8^ Department of Respiratory Medicine, Hannover Medical School and Biomedical Research in End-stage and Obstructive Lung Disease Hannover, German Lung Research Center (DZL), Hannover, Germany; ^9^ Laboratory of Hepatology, CHROMETA Department, KU Leuven, Leuven, Belgium; ^10^ Department of Gastroenterology and Hepatology, UZ Leuven, Leuven, Belgium

**Keywords:** lung transplantation, chronic rejection, imaging, single-cell profiling, mouse model

## Abstract

**Introduction:**

Chronic rejection is a major complication post-transplantation. Within lung transplantation, chronic rejection was considered as airway centred. Chronic Lung Allograft Dysfunction (CLAD), defined to cover all late chronic complications, makes it more difficult to understand chronic rejection from an immunological perspective. This study investigated the true nature, timing and location of chronic rejection as a whole, within mouse lung transplantation.

**Methods:**

40 mice underwent an orthotopic left lung transplantation, were sacrificed at day 70 and evaluated by histology and in vivo µCT. For timing and location of rejection, extra grafts were sacrificed at day 7, 35, 56 and investigated by ex vivo µCT or single cell RNA (scRNA) profiling.

**Results:**

Chronic rejection originated as innate inflammation around small arteries evolving toward adaptive organization with subsequent end-arterial fibrosis and obliterans. Subsequently, venous and pleural infiltration appeared, followed by airway related bronchiolar folding and rarely bronchiolitis obliterans was observed. Ex vivo µCT and scRNA profiling validated the time, location and sequence of events with endothelial destruction and activation as primary onset.

**Conclusion:**

Against the current belief, chronic rejection in lung transplantation may start as an arterial response, followed by responses in venules, pleura, and, only in the late stage, bronchioles, as may be seen in some but not all patients with CLAD.

## Introduction

Lung transplantation is a life-saving treatment for end-stage lung diseases. However, the lung is prone to rejection due to the strong allo-immune response of the specialized mucosal immune barrier of the lung epithelium. Rejection represents the Achilles’ heel of lung transplantation, with a survival rate below that of other solid organ transplantations (5-year survival of 59%) (1). Transplant immunologists have classified rejection into three stages depending on the timing post-transplant: hyperacute, acute, and chronic rejection (2), occurring within the first hours, weeks, or more than 6 months after transplantation. Chronic rejection involves cellular and humoral immune activation, is poorly responsive to treatment, and consequently is the main culprit for long-term survival (2). The clinical presentation of chronic rejection is a gradual late allograft dysfunction in which other causes such as infection and malignancy are excluded (3). Pathologically, chronic rejection in organ transplantation is characterized by vascular intimal thickening and fibrosis, resulting in graft necrosis, atrophy, and loss of functionality. In lung transplantation, the destruction of only small airways, pathologically termed obliterative bronchiolitis (OB), was considered the manifestation of chronic rejection (4). Chronic rejection is presumed to be the immunological counterpart of the clinical concept of chronic lung allograft dysfunction (CLAD), uniting all late persisting lung function deteriorations without identifiable cause (3, 5). Understanding how the immunological concept of “specific” rejection fits into the clinical concept of “non-specific” CLAD [bronchiolitis obliterans syndrome (BOS) and restrictive allograft syndrome (RAS)] is essential in determining the true nature of clinical rejection, resulting in better patient management and outcome.

The mouse orthotopic left lung transplant model based on the cuff technique (6, 7) is a unique way to study rejection. This model involves all essential elements to properly study lung transplant rejection “in a controlled way”, as it includes the lung as a functioning organ, an immune response responding against an MHC (H2) mismatch, the role of immunosuppression on the lung "architecture", and immune system, and “secondary immunodeficiency”. Reports on chronic rejection in orthotopic lung transplantation mostly involved a minor mismatch setting without immunosuppression (8–10); however, we developed a unique model of chronic rejection combining a major genetic mismatch with daily immunosuppression (11, 12).

Our aim was to document the true nature of the immune system “rejecting” the foreign donor lung within a controlled mouse lung transplant setting. This study addresses the timing, the location, and the different elements of the immune system (innate and adaptive) and lung (airways, vessels, parenchyma, and pleura) changes during chronic rejection by using histology, *in vivo* and *ex vivo* μCT imaging, and single-cell RNA profiling.

## Methods

### Mouse orthotopic left lung transplantation

All mice received human care in compliance with the European Convention on Animal Care and the *Guide for the Care and Use of Laboratory Animals* published by the National Institutes of Health (NIH publication 86-23, 1996). The study was approved by the Ethics Committee for Animal Research at KU Leuven (P008/2017). Male C57BL6/N and BALB/C mice, 10–12 weeks old, were purchased from Janvier Labs (France). Orthotopic left lung transplantation was performed as described by Jungraithmayr et al. (7). In summary, following thoracotomy, the artery, vein, and bronchus were separated from each other, and 10-0 ligatures were placed around the structures. The pulmonary artery and pulmonary vein were closed using 9.0 sutures. First, the vein was anastomosed, followed by the artery, and finally the bronchus. The sutures were released and the lung was inflated. Hereafter, the chest was closed, and the animals were placed on a heating pad after waking up.

### Post-transplant study design

A total of 80 mice, consisting of 52 C57BL6/NRj and 28 BALB/cJRj mice, were used. In total, 28 left lung allografts from BALB/c donor mice were transplanted into C57BL/6N recipients. Twelve isograft transplantations were performed with C57BL/6N donor lungs in C57BL/6N recipients as controls. All mice received daily maintenance immunosuppression subcutaneously, consisting of cyclosporine (10 mg/kg/d CsA; Novartis, Belgium) and steroids (1.0 mg/kg/d or 1.6 mg/kg/d methylprednisolone; Pfizer, Belgium). The low dose of steroids is equivalent to the human situation, and the high dose corresponds to other mouse models considering the higher metabolism of mice (11, 12).

**Figure 1:**
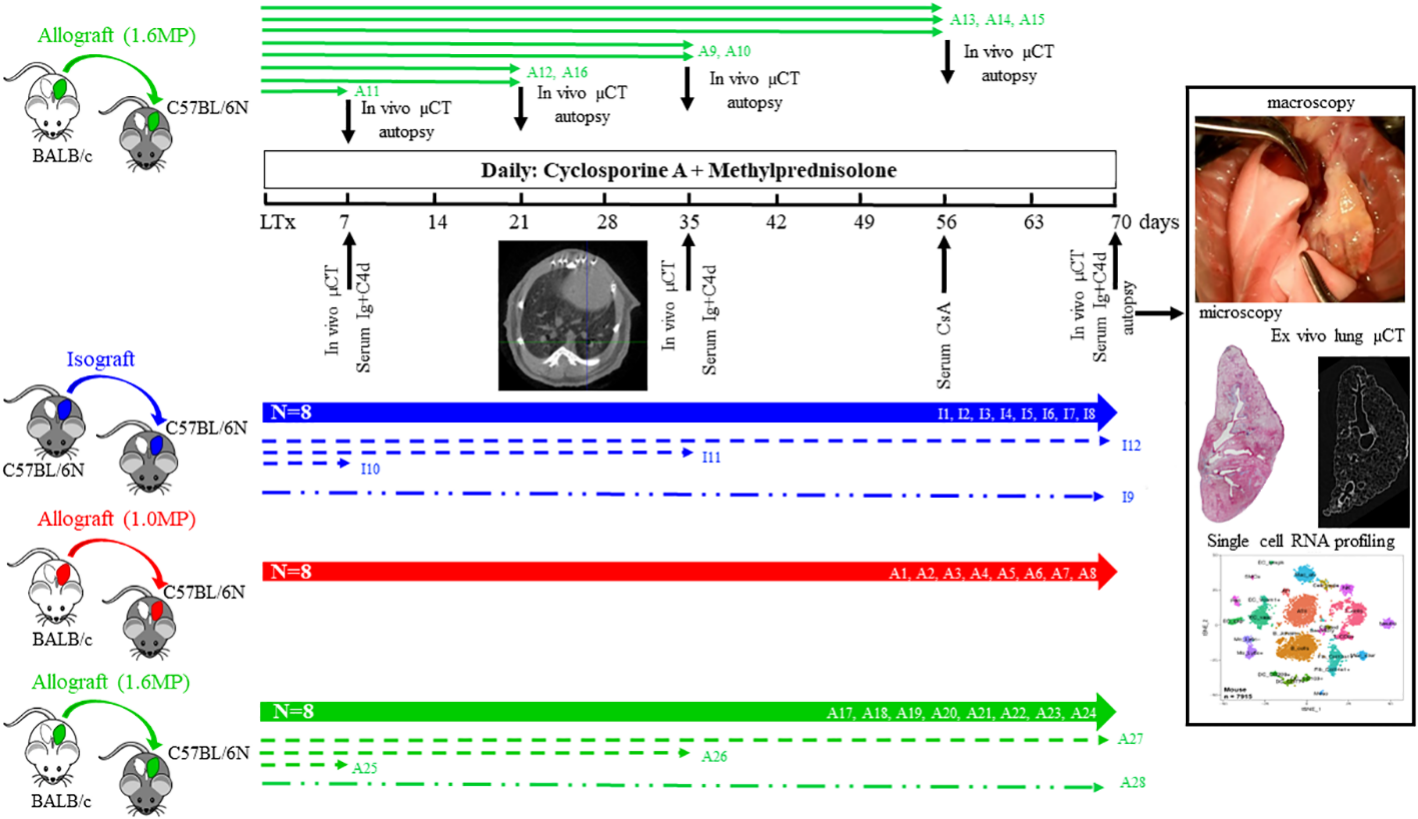
The study design of the allograft and isograft orthotopic single left lung transplantation in mice receiving daily immunosuppression of cyclosporine and steroids. Isograft (blue), allograft (low dose of steroids; red), and high dose of steroids (green) were sacrificed at 10 weeks (*n* = 8/group; thick lines). Additional allografts (high dose; *n* = 8) are sacrificed at weeks 1, 3, 5, and 8 (green, thin lines). Evaluation parameters post-transplantation are *in vivo* lung imaging, serum sampling and histology, *ex vivo* lung imaging, and single-cell analysis. Additional mice for single-cell RNA profiling and the *ex vivo* µCT are presented as dotted lines and dot-dashed lines. All animals are coded and reported later on.

**Figure 2:**
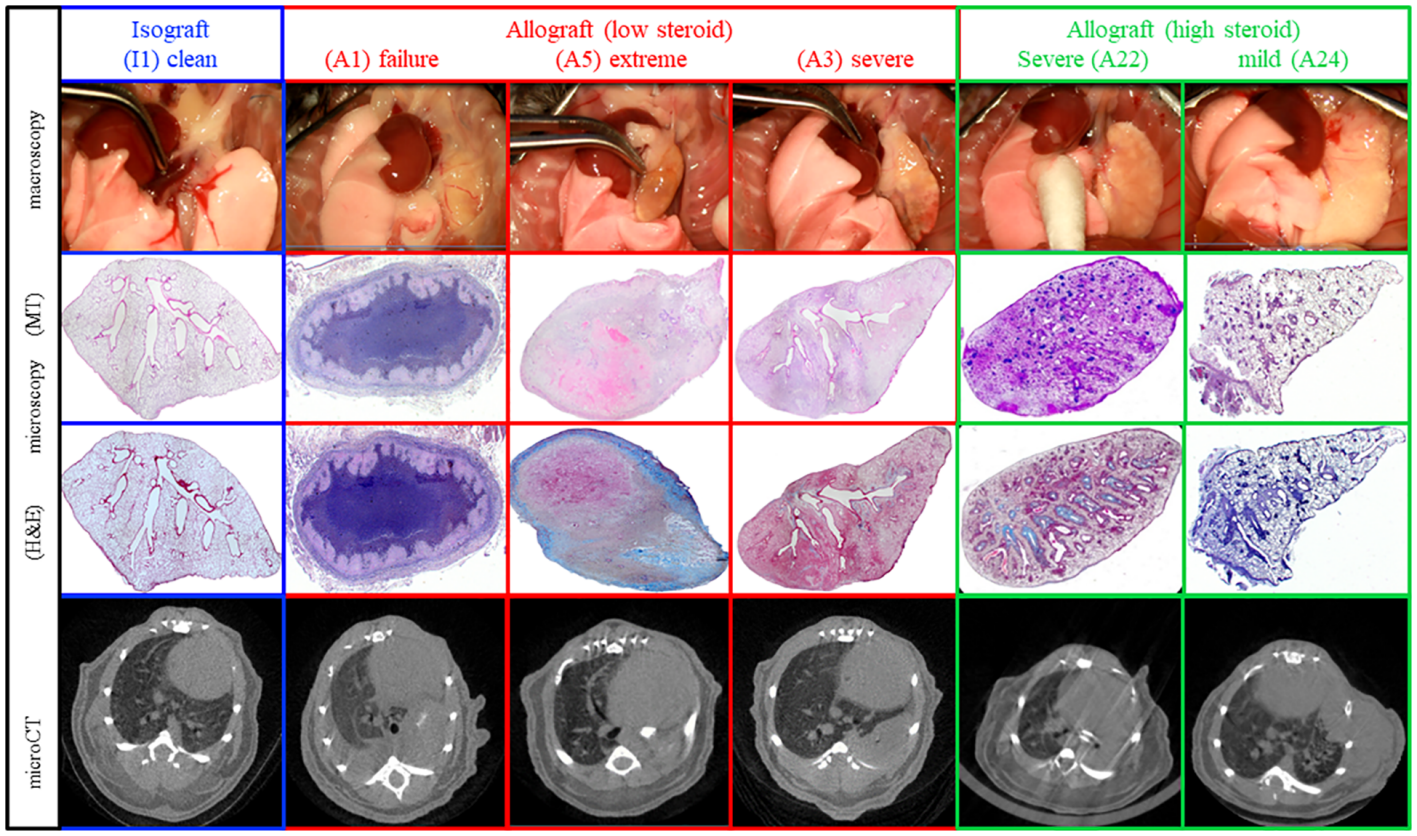
Representative macroscopy, microscopy, and *in vivo* µCT of the different pathological presentations at day 70. The different patterns include fully normal lungs, completely destroyed failures, and lungs demonstrating chronic rejection with a spectrum of extreme, severe, and mild rejection.

**Figure 3:**
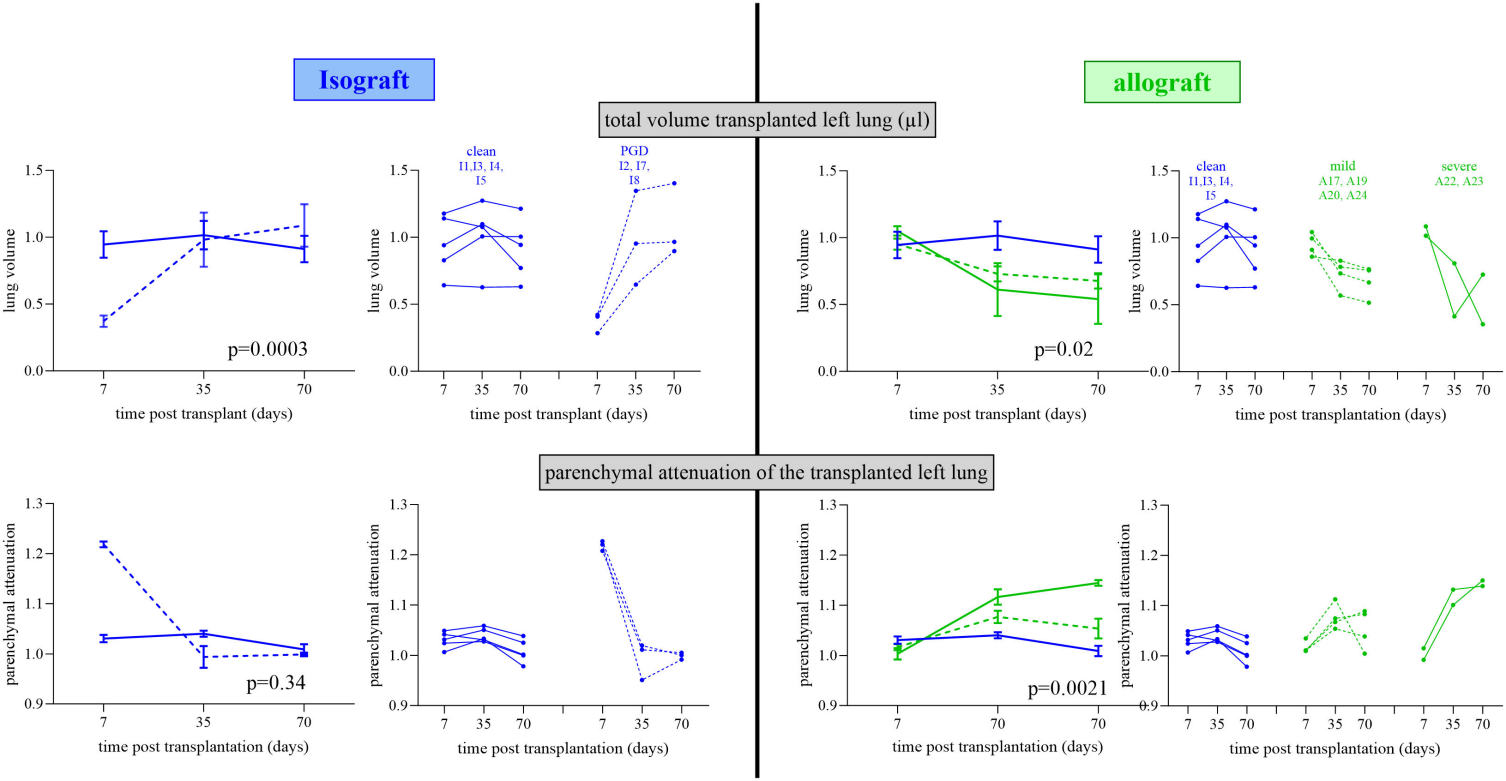
Repeated *in vivo* µCT lung evolution of the isograft and allograft groups. *In vivo* µCT lung evolution for lung volume and parenchymal attenuation. µCT parameters are normalized to the reference lungs. The left side shows the isografts (blue lines) stratified according to the occurrence of PGD (dotted line) or not (full line) with group variation and individual evolution. The right side shows isografts (no PGD; blue) and allografts (green) stratified according to mild (dotted line) and severe (full line) rejection with group variation and individual evolution. For group variation, the median with SEM is presented at each time point.

In the first set of transplantations, 16 allografts and 8 isografts were monitored daily until sacrifice (day 70). The 8 isografts (I1–I8) and the first 8 allografts received an immunosuppression regimen of CsA with low steroids (A1–A8) (*n* = 8), while the second 8 allografts received CsA with high steroids (A17–A24). The follow-up included the following: daily body weight monitoring, cyclosporine measurement in the blood (retro-orbitally bleeding) at day 56, blood sampling to measure immunoglobulins and complement, and *in vivo* µCT imaging at days 7, 35, and 70 (**Figure 1**). At sacrifice, a video of the ventilating lung was recorded to document the lung functionality. The macroscopic status of the lungs was classified as failure, extreme deformation, severe deformation, and mild changes (**Figure 2**). A failed lung had shrunken and was non-ventilating, with or without attachment to the thoracic wall. Within extremely deformed lungs, the lung structures (vessels, airways, and parenchyma) could not be discriminated against anymore, and only a hard, solid fibrotic mass was observed on histology. Within severe deformation, lung structures could still be identified, but no ventilation of the lung was seen. Macroscopically, mildly rejecting lungs were still ventilating and had a normal volume and normal surface appearance (**Figure 3**, **Supplementary Figure S3**). In a second group of eight allografts receiving CsA and high steroids, mice were sacrificed earlier to investigate the macroscopic changes and microscopic presentation of the early µCT. Allografts were sacrificed at day 7 (A11; *n* = 1), day 21 (A12 and A16; *n* = 2), day 35 (A9 and A10; *n* = 2), and day 56 (A13–A15; *n* = 3).

A last set of transplantations of three isografts (I10–I12) and three allografts (A25–A27) were sacrificed at days 7, 35, and 70, and the transplanted lungs were used for single-cell RNA sequencing. One healthy/untreated BALB/cJRj and one healthy/untreated C57BL/6NRj left lung were used as baseline controls. Finally, for *ex vivo* imaging, one isograft (I9) and one allograft (A28) were sacrificed at day 70. An overview of the mice and methodology is presented in **Figure 1**.

### Longitudinal *in vivo* µCT imaging

To evaluate the left transplanted lung during follow-up, *in vivo* µCT imaging (days 7, 35, and 70) was performed with a small-animal µCT scanner (SkyScan 1278, Bruker, Belgium; resolution = 55 µm³). Mice were anesthetized, and respiratory-gated µCT images of free-breathing animals were acquired. The respiratory cycle was divided into four phases, from the initiation of inspiration to end expiration, and scan parameters were described previously (13) (14) to quantify lung volume and mean lung density for a manually delineated volume of interest (VOI) on the transversal µCT images at end expiration (15). The left transplanted and right native control lungs were analyzed separately to investigate their changes properly. To make the data comparable, untreated C57BL/6N (*n* = 4) and BALB/c (*n* = 4) littermates were scanned to create baseline values. Owing to the anatomical differences in lung structure and volume, transplanted lungs of isografts were normalized using C57BL/6N baseline data, and transplanted lungs of allografts were normalized using BALB/c baseline data.

### Lung histopathology

Lungs were fixed (10% formalin, 24 h) and paraffin sections (7 μm) were stained with hematoxylin–eosin (H&E) and Masson trichrome (MT), and sections were evaluated by a pathologist to identify the pathological elements of chronic rejection. To observe how lung structural changes parallel the organization of the immune system, mice were sacrificed at different time points to find a sequence of events with respect to lung architecture, as performed previously (11, 12).

### *Ex vivo* µCT

One isograft (I9) and one mildly rejecting allograft (A28) lung collected at day 70 were used for *ex vivo* µCT to reconstruct the early changes in an allograft. The grafted lung was fixated (10% formalin; 24 h), followed by ethanol dehydration (70%/80%/90%/100%) and complete chemical drying in hexamethyldisilazane. Dried lungs were scanned using an *ex vivo* SkyScan 1272 µCT scanner (resolution = 2.5 µm; Bruker) to segment the airway, veins, and arterial lumen by ITK-SNAP (16).

### Blood analysis

Cyclosporine blood levels were analyzed with an immunoassay (Dimension^®^ RXL, Diamond Diagnostics, USA). Serum immunoglobulins (IgGA/IgE/IgM/IgG1/IgG2b/IgG2c/IgG3) were measured with a ProcartaPlex Mouse Isotyping Panel (Thermo Fisher, Belgium). Serum complement factor 4d was measured by the conventional ELISA kit C4d (MyBioSource, USA).

### Single-cell RNA sequencing

Grafted lungs from three isografts (I10–I12) and three allografts (A25–A27) at days 7, 35, and 70 were excised and immediately processed into single-cell suspension according to the Miltenyi protocol (Miltenyi Lung Dissociation Kit mouse). One BalBc lung was included as a comparison. Single-cell suspensions of the left lung of seven mice (control BALB/c, *n* = 1; isografts at days 7 and 70, *n* = 1 per time point; allografts at days 7, 35, and 70, *n* = 1 per time point) were successfully obtained. Briefly, the lungs were flushed, excised, and cleaned of excess tissue. The MACS enzyme solution was instilled into the lung, and the lobes were transferred into gentle MACS tubes containing the enzyme mix to dissociate cells. Single-cell suspensions were cryopreserved in liquid nitrogen until sequencing. Single-cell RNA sequencing was performed using the 10x Genomics 3-prime-v3 dual index assay using the manufacturer’s protocol. Sequencing was performed using an Illumina HiSeq4000. Read alignment was made as previously published (17) using the mouse genome (GRCm39). The gene-cell matrix was inputted into Seurat (v4.0.3) for analysis. The matrix was filtered to remove cells with <1,000 reads or >5% mitochondrial genes, normalized, and scaled with a regression of mitochondrial gene percentage. Clusters were grouped using Louvain clustering, and cell-type clusters were determined using canonical marker genes and FindAllMarkers to identify uniquely expressed genes based on their expression of these marker genes. Cells were then classified into epithelial, endothelial, stromal, and immune groups based on their type. UMAP reduction was used for visualization. Differentially expressed genes were identified using the FindMarkers set to compare allograft time points (e.g., A1W) with all other groups. GO enrichment analysis was performed with *clusterProfiler* (v3.18.0) (18). Up- and downregulated genes of allografts compared to isografts and controls were identified using the *FindMarkers* function in *Seurat* (expression in at least 10% of cells, adjusted *p*-value < 0.05, and average log2 fold change < −0.25 or >0.25). GO enrichment analysis was performed using enrichGO with default parameters and the org.Mm.eg.db (v3.12.0). Revigo and simRel were used to summarize GO ontology terms (19). Adjusted log10 *p*-values were visualized using *ggpubr* (v0.4.0). Connectome analysis was performed using the Connectome (v1.0.0) package on github (https://msraredon.github.io/Connectome/). Default parameters were used with the exception of setting the minimum *Z*-score to 2.6 for visualization. Cellular archetypes were identified with pseudotime analysis using the phateR (v1.0.7) and slingshot (v1.8.0) packages in R to determine cells that show correlated or unique features with disease progression. The visualization of heatmaps was done using ComplexHeatmap (v2.6.2).

### Statistics

Data analysis was performed with Prism10 (GraphPad, USA) and expressed as the mean ( ± SEM). The D’Agostino and Pearson normality test was performed. To compare the different groups, a one-way analysis of variance (ANOVA) was used, and to compare different groups over time, a mixed-effects model with Tukey’s multiple comparisons post-hoc test was used. A Mann–Whitney *U* test was performed to compare the different transplant groups and time effects. A value of 0.05 was considered significant.

## Results

### Macroscopic and microscopic evaluation of the lung grafts

**Figure 4:**
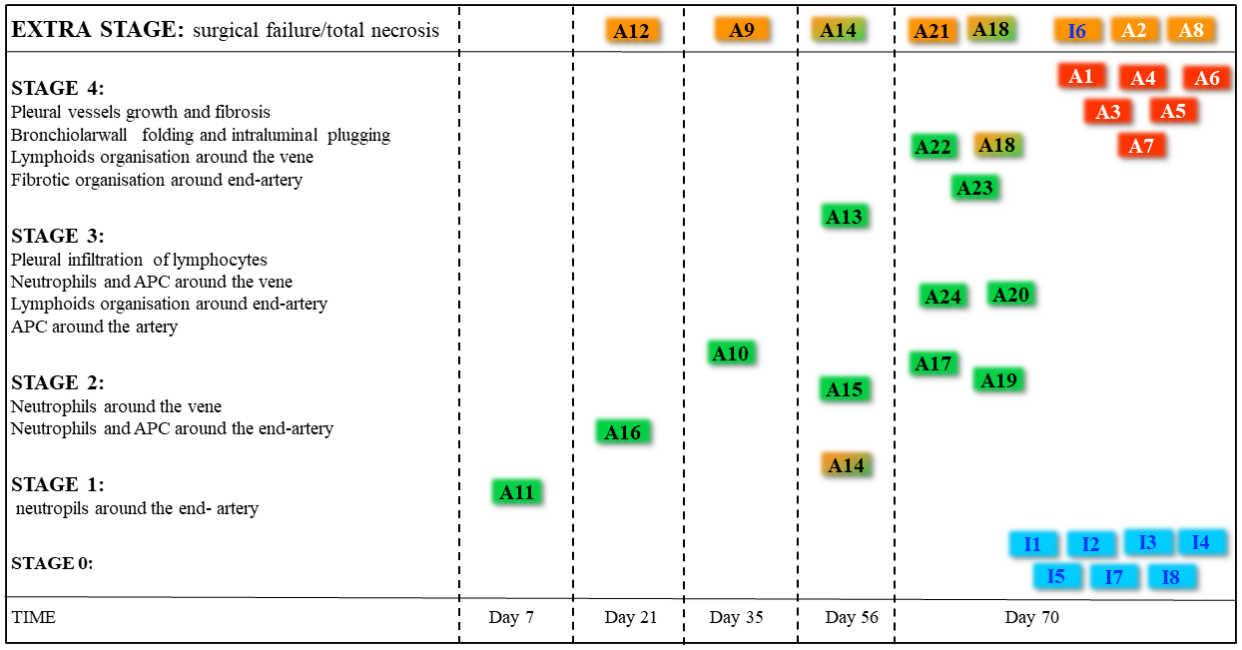
The pathological staging of rejection. All allografts were used to identify the stage and were subdivided into stages of rejection. Failures are documented in orange boxes. Two animals demonstrated a part of the lung to be destroyed, and another part presented rejection (half green half orange boxes). The color of the boxes in the included lungs represents the origin of the graft being an isograft (blue, n = 7), an allograft under high-dose steroids (green boxes, n = 13), and an allograft under low-dose steroids (red boxes, n = 6).

Macroscopic evaluation of the transplanted lungs revealed differences across and within different groups (**Figure 2**, **Supplementary Figure S1**). Evaluation of the 24 transplanted lungs at day 70 revealed five failures, including one isograft (I6), two allografts with low steroid (A2 and A8), and two allografts with high steroid (A18 and A21). Failures histologically presented necrosis destroying the lung, including parenchyma, airways, and vessels. In three of the graft failures (I6, A1, and A8), an end-stage fibrotic mass with few cells was the only remaining fragment of the lung. To properly study chronic rejection, failures were excluded from further analysis. Macroscopically, all isografts had a normal lung color and morphology and were ventilated well. Within the low-steroid allografts, three allografts showed extreme deformation (A1, A4, and A6), and three allografts showed severe deformation (A3, A5, and A7). Within the high-steroid group, two allografts (A22 and A23) were severely deformed, while four allografts (A17, A19, A20, and A24) were mildly affected. Within the allografts, only mild rejecting allografts were ventilating at day 70 (**Supplementary Figure S1**). There were no differences in body weight (*p* = 0.92) and cyclosporine levels (*p* = 0.35) between the three groups (**Supplementary Figure S2**). The cyclosporine level of all mice was 587 ± 35 µg/L. Microscopic evaluation supported the macroscopic observation (**Figure 4**). Macroscopically, extreme deformation within the low-steroid allografts (A2, A4, and A6) was presented as intense end-stage fibrosis in all compartments. Although the vessels and airways could be located, these were never functional.

### *In vivo* repeated μCT evaluation

**Figure 5:**
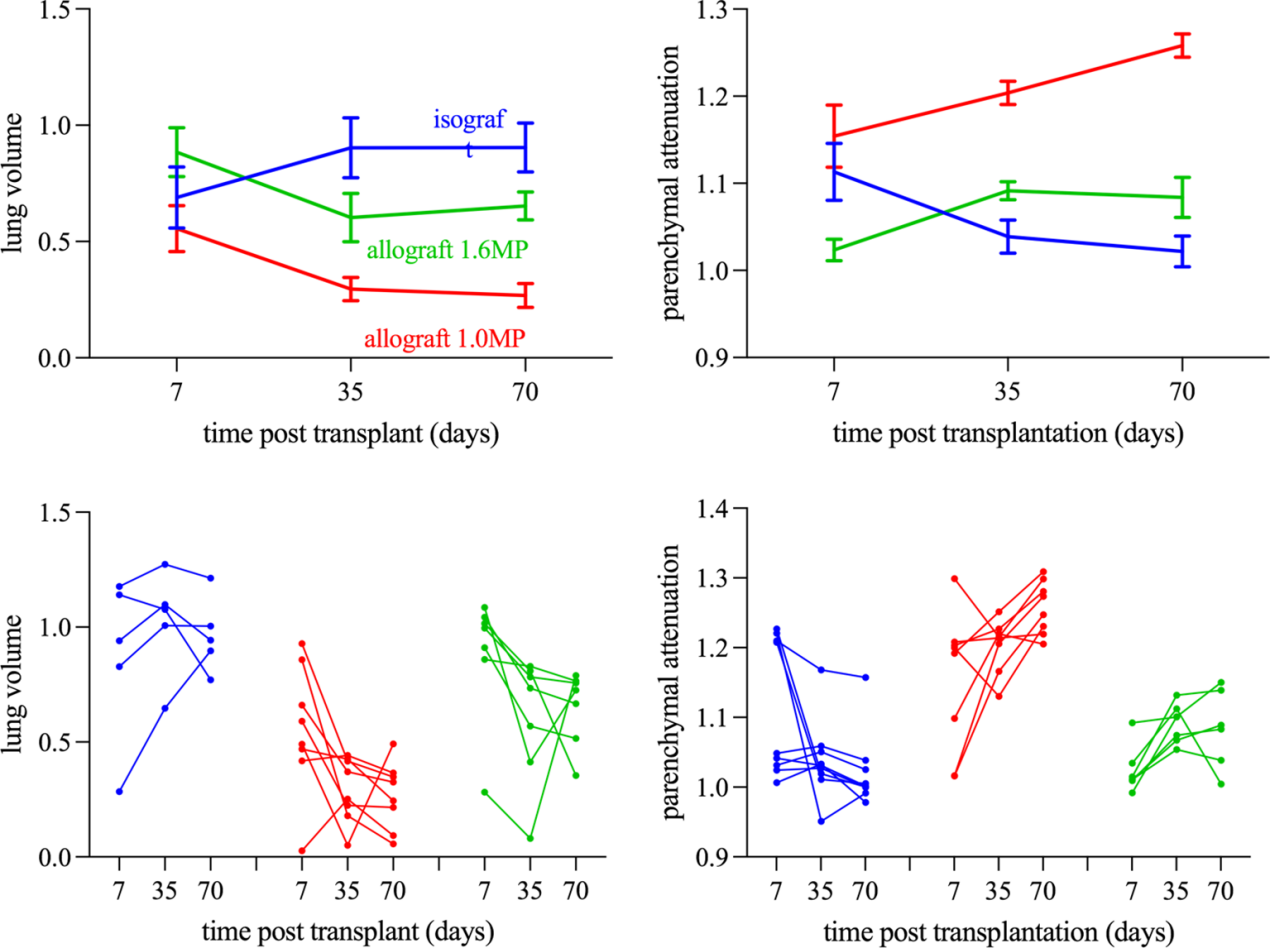
Longitudinal morphometric µCT analysis stratified according to the severity of rejection (Tx left lung). **(A)** Total lung volume of the transplanted left lung. **(B)** Parenchymal attenuation of the transplanted left lung. The Left shows the isograft group (blue lines) stratified according to the occurrence of PGD (dotted lines). The right shows the Allograft 1.6MP group (green lines) stratified according to the severity of rejection (mild rejection is denoted as dotted lines and severe rejection is denoted as full lines). All data have been normalized as described in the Methods section.

**Figure 6:**
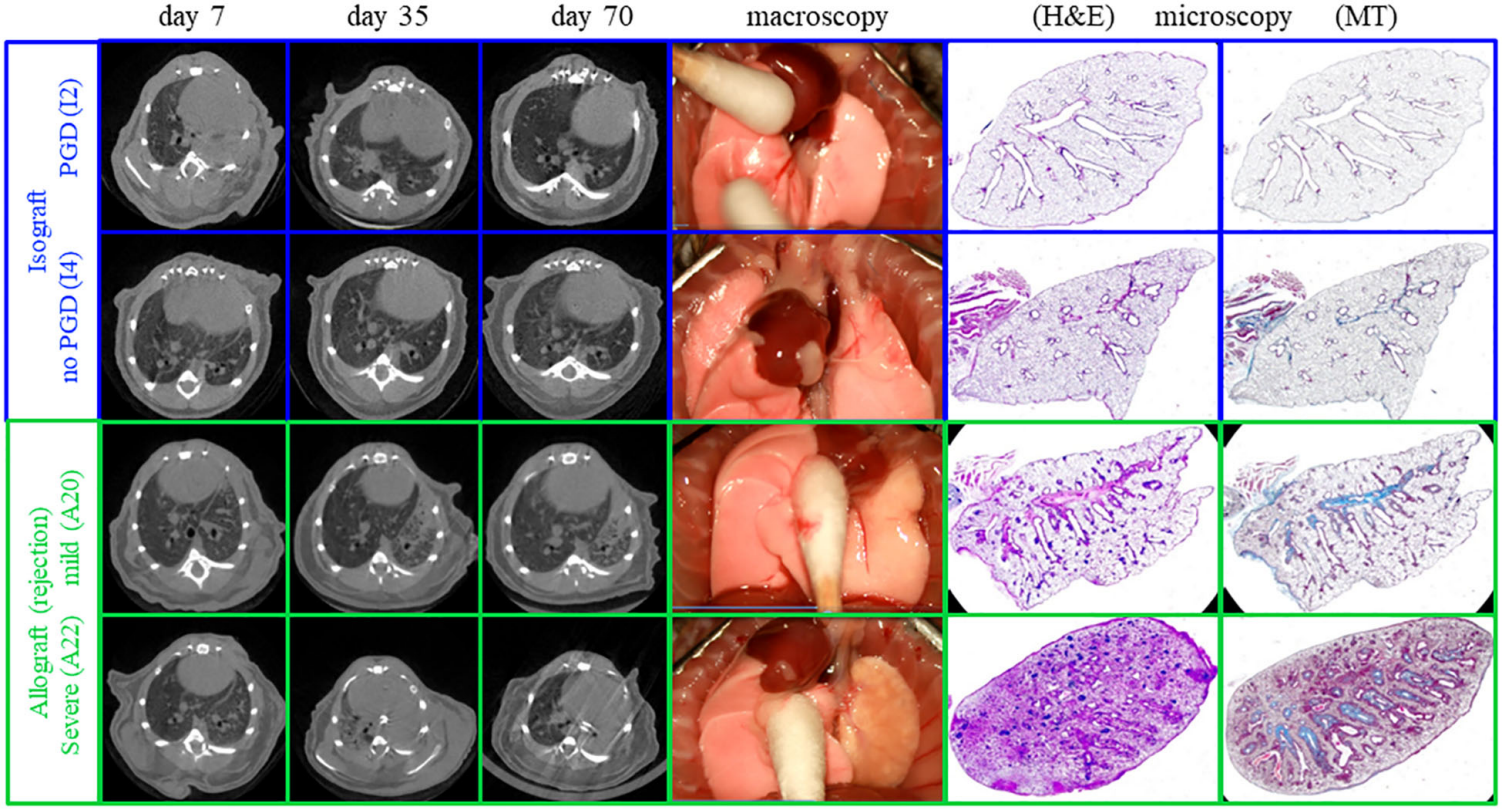
Representative macroscopy, microscopy, and *in vivo* µCT imaging of mild and severe chronic rejection. One allograft with mild rejection and one with severe rejection are presented (green). In comparison, a control isograft is present, and an additional isograft demonstrating on µCT at week 1 primary graft dysfunction (PGD).

Evaluation of lung volume changes showed differences within isografts and allografts over time (*p* < 0.0001) (**Figure 5**). Lung volume at day 7 was comparable between allografts and isografts. Over time, isografts showed an increasing lung volume, while allografts decreased. Changes in lung volume were observed between mildly and severely affected allografts versus clean isografts (*p* = 0.025), as mildly and severely rejected allografts had volume reductions at day 35 (*p* = 0.12 and *p* = 0.39) and day 70 (*p* = 0.18 and *p* = 0.40) versus isografts (**Figure 5**). The lung volume difference between inspiration and expiration on μCT scans (a type of tidal volume) was compromised in allografts and was greatly compromised in severely rejecting allografts. Analysis of the right native lung confirmed that the decrease in tidal volume was caused by the graft (**Supplementary Figure S3**). Parenchymal attenuation of the transplanted lung was different (*p* < 0.0001) between isografts and allografts. Allografts were initially open (day 7) and lung attenuation appeared afterwards (**Figure 6**). Attenuation was increased in mild and severe rejecting allografts at day 35 (*p* = 0.087 and *p* = 0.036) and day 70 (*p* = 0.15 and *p* = 0.0002) versus isografts. Low-steroid allografts had more attenuation than high-steroid allografts at days 35 and 70 (*p* = 0.0011 and 0.009). The native lung had no increased attenuation, confirming the absence of collateral damage or possible infection (**Supplementary Figure S4**). Isografts demonstrated a normal lung appearance without attenuation at day 70, but at day 7, some isografts demonstrated attenuation and resembled potential primary graft dysfunction (PGD) (**Figure 3**). PGD is a type of severe lung injury that occurs within the first 72 h of lung transplantation and is the most common cause of early mortality. PGD decreased towards days 35 and 70. PGD is a graft defect. Repeated μCT revealed lung volume differences between isografts with and without PGD (*p* = 0.0003). While the lung volume in PGD was lower at day 7, it returned to the level of the isografts without PGD at days 35 and 70 (**Figure 3**).

### Pathological pattern of chronic rejection in time and space

**Figure 7:**
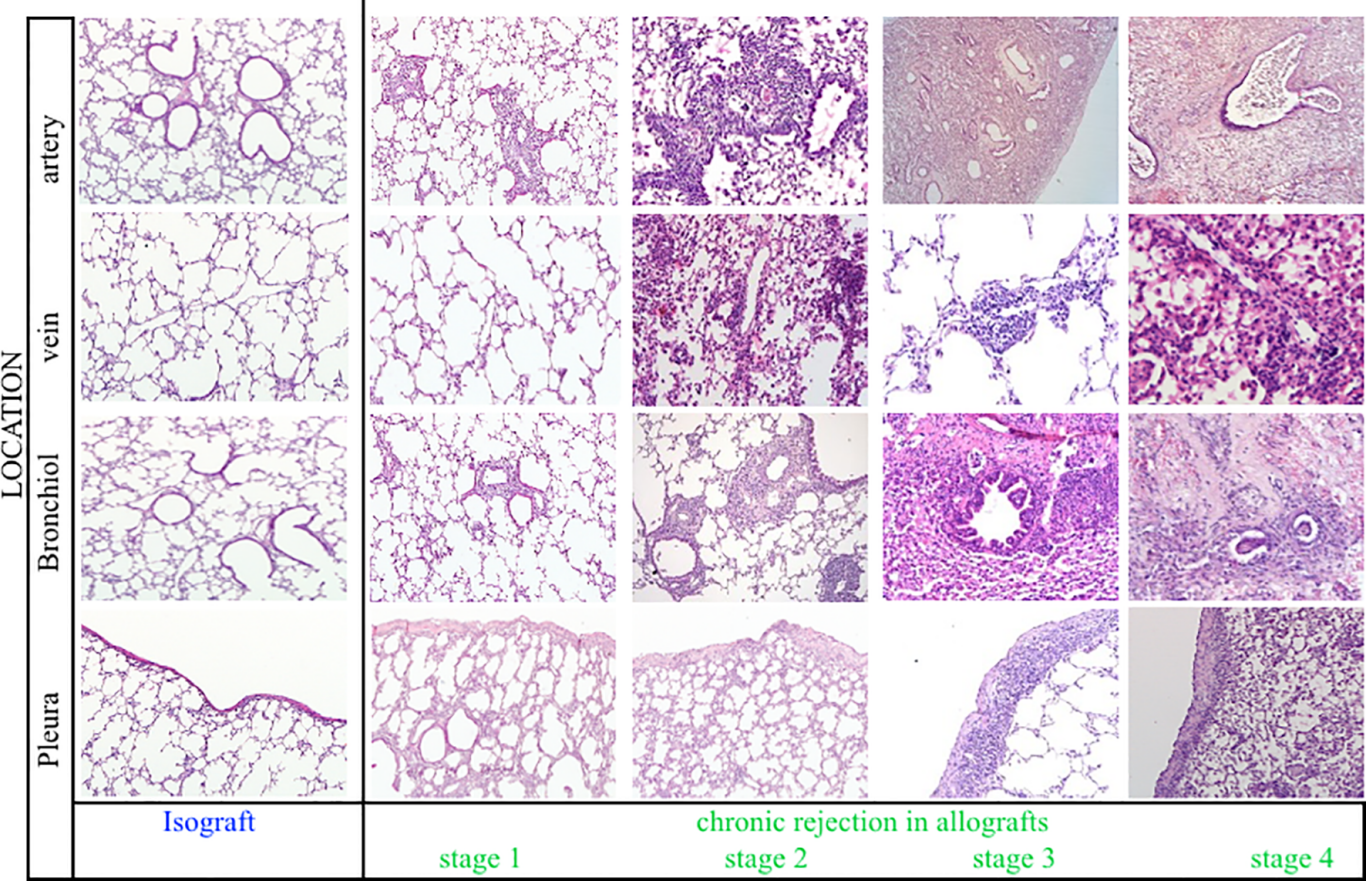
Representative histological illustrations of the four stages of chronic rejection. For each stage, the different anatomical lung compartments involved were presented, including arteries, veins, bronchioles, and pleura. Stage 1, Stage 2, Stage 3, and Stage 4 are represented by A11 at day 7, A16 at day 21, A24 at day 70, and A22 at day 70, respectively.

**Figure 8:**
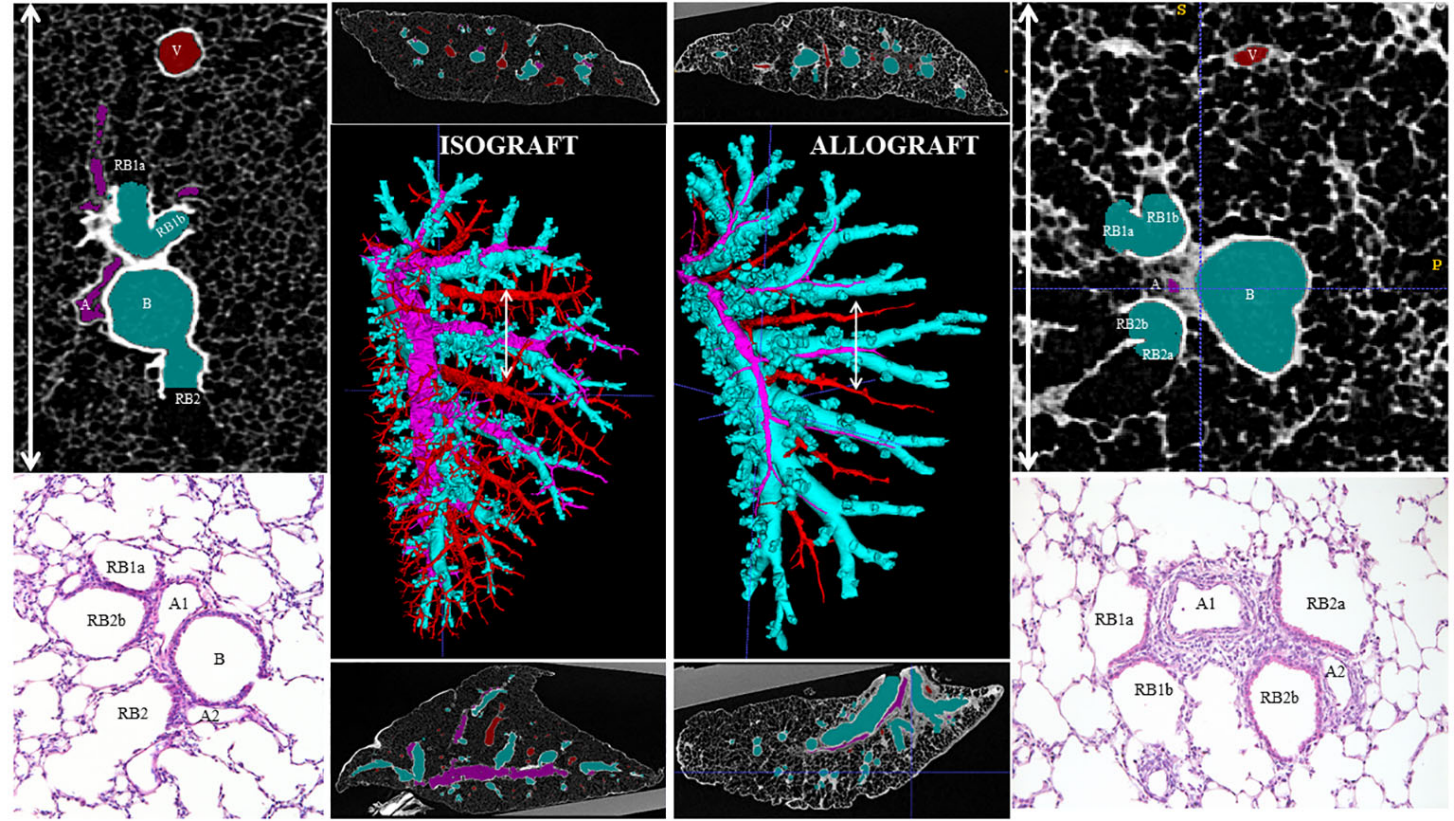
An *ex vivo* high-resolution µCT imaging and reconstruction of the organization of chronic rejection. The airways (light blue), arterial vessels (pink), and venous vessels (red) of the transplanted left lung were segmented and reconstructed in 3D (middle large picture) in an isograft (left) and an allograft (right). A transverse and sagittal image of the scans is presented above and below the reconstruction. On the right and left sides of the figure, μCT (top) and histological (bottom) details of the broncho-vascular bundle, specifically of the location of the white arrow line, identify the arterial origin of rejection at the generation where airways go over in respiratory bronchioles.

Pathological examination of allografts under high immunosuppression revealed an evolutionary pattern of chronic rejection organized by time, location, and immune response (**Figure 7**). Stage 1, shown in an allograft (A11, day 7), presented neutrophil extravasation into the vessel wall of end-arterioles, reducing the arteriolar lumen without increasing wall thickness. Stage 2 showed inflammation around end-arterioles and end-venules. The innate activation around end-arterioles increased in size with the influx of antigen-presenting cells. Simultaneously, neutrophils infiltrated the end-venules, but the bronchioles were still not involved. *Ex vivo* µCT confirmed the vessel lumens narrowing for both arteries and veins, while the airway lumen remained unaffected (**Figure 8**, **Supplementary Figure S5**). Evolution toward Stage 3 consisted of immune organization around arteries evolving toward adaptive activation. Venules remained innate, but monocytes appeared besides neutrophils. The lumen of the venules decreased, and the pleural compartment started to be infiltrated by lymphocytes. The final stage of rejection (Stage 4) of arterioles evolved toward fibrosis with end-arterial obliterans. The venous compartment remained innately immune-organized, and bronchioles affected by the neighboring arteriolar inflammation demonstrated typical bronchiolar folding, but only one allograft presented fibrotic intraluminal plugging (bronchiolitis obliterans).

### Single-cell RNA profiling to validate the sequence of chronic rejection

**Figure 9:**
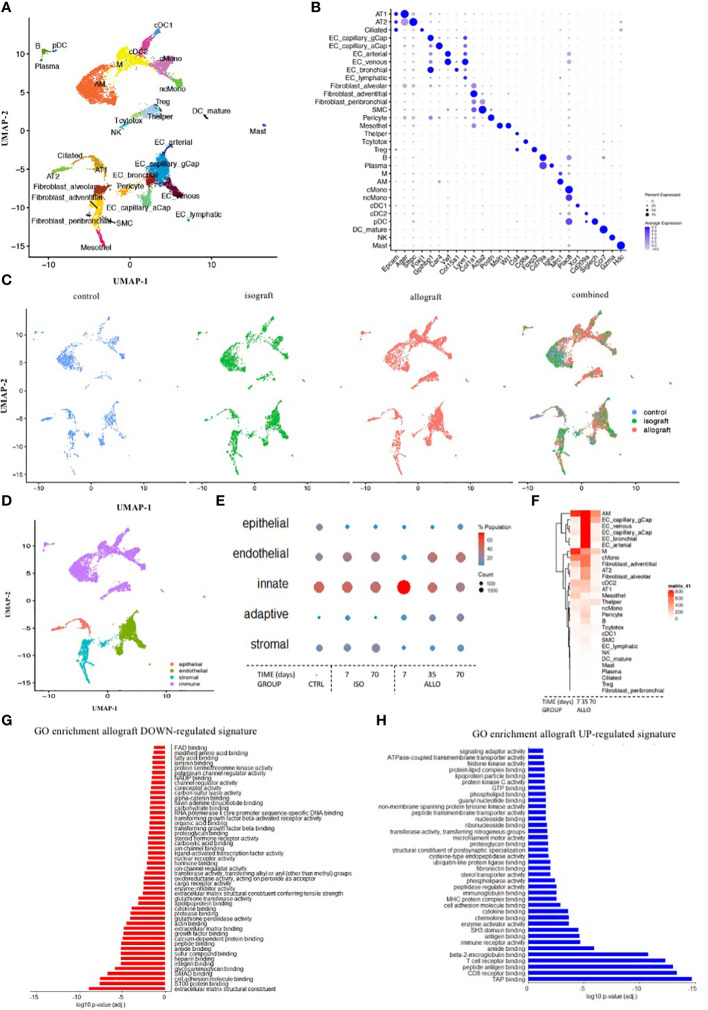
Single-cell RNA profiling to validate the sequence of chronic rejection across the different cells involved and subcellular mechanisms. **(A)** UMAP plot of the cells from the left lung of three isografts (7, 35, and 70 days), three allografts (7, 35, and 70 days), and one control BalBc lung color-coded by major cellular lineage. **(B)** Dot plot heatmap of the expression of representative marker genes of cellular lineages. The size and color intensity of each dot represent, respectively, the percentage or average expression of the marker gene in this cell type. Color scale: blue, high expression; white, low expression. **(C)** UMAP plot of lung cells, color-coded for the indicated conditions of the left lung. **(D)** UMAP plot of lung cells, color-coded for the indicated major cell subcluster. **(E)** Dot plot heatmap of the major cell subcluster. The size and color intensity of each dot represent, respectively, the total number and percentage of cells within each cell type. Color scale: red, high expression; blue, low expression. **(F)** Gene expression heatmap of all individual genes in every identified cell type. Color scale: red, high expression; blue, low expression. **(G, H)** A barplot of the GO enrichment analysis of down- and upregulated gene signatures in allograft lungs.

Single-cell profiling sequenced 12,821 cells from seven mice. Each specific cell within control, isograft, and allograft lungs was presented in a UMAP plot and gene marker validation (**Figures 9A-C**). The different cell types were clustered into structural epithelial/endothelial, innate, adaptive, and stromal cells (**Figures 9D, E**). The structural cells decreased at day 7 in allografts compared to isografts and controls and recovered gradually at days 35 and 70 (**Supplementary Figure S7C**). Innate cells increased at day 7 and returned to the isograft and control levels at days 35 and 70 (**Figure 9E**, **Supplementary Figures S7A-C**). Cell profiling of eosinophils and neutrophils was unsuccessful and could not be evaluated. Adaptive cells, including T helper and cytotoxic cells, B cells, plasma B cells, and Treg cells, gradually increased toward day 70. Finally, stromal cells started to increase and eventually generated the fibrotic environment (**Figure 9E**) for chronic rejection. Although adventitial fibroblasts appear to be the leading producers of extracellular matrix (ECM), multiple stromal cells were upregulated.

Overall gene expression in allografts was increased versus isografts and control lungs. Endothelial cells and monocytes/macrophages showed a particular increase in gene expression, suggestive of a key role in the onset and progression of rejection (**Figure 9F**). Upregulation of signaling pathways linked to the immune response, with innate and adaptive elements such as MHCI/II elements, receptor binding, proteasome formation, and downstream signal transduction (**Figure 9H**), was found. In the early onset of rejection, macrophages presented an increased expression of MHCII together with CXCR3, CXCL14, and FcRE. T cell receptor-related costimulatory elements are upregulated at an early stage, even prior to the increase and proliferation of the T cells (**Supplementary Figure S8D**). To examine the temporal evolution of the expression, pseudotime analysis identified genes involved in early and late processes (**Supplementary Figures S8E, F**). Within the Tc cells, inducer and effector cytokines are increasingly expressed (**Supplementary Figure S8C**). ECM production was very active in the early period after transplantation and subdued at day 70 (**Supplementary Figure S9D**). Contractile properties are present in smooth muscle cells, whose expression levels decreased early after transplantation of isografts but recovered later (**Supplementary Figure S9D**). Connectome analysis confirms these complex changes of lung homeostasis and immune activation with innate cells linked to endothelial cell involvement early on and to adaptive cell involvement later on (**Supplementary Figures S9E, F**). GO enrichment analysis confirmed that structural cells and stromal cells have very low general gene expression. Downregulation of signaling pathways related to cell homeostasis, integrity, and organization was observed to be involved in the onset of rejection (**Figure 9G**). Mechanistic clues demonstrated that isografts receiving immunosuppression had lower levels of MHC molecules in the structural cells. However, during rejection, MHC expression increased in allografts above isograft levels (**Supplementary Figure S7D**). These higher levels of MHCI are confirmed by the increase in proteasome elements, expression of chemokines, and interferon elements (**Supplementary Figure S7E**). In addition, MHCII expression was also increased, especially during the early phase of rejection.

### Systemic humoral involvement

Measurements of humoral components showed large inter-individual variation, making it difficult to reach significance. IgA and IgG1 were below detection, while immunoglobulins IgG1, IgG2, and IgG3 tended to increase in severe rejection. IgG2c and IgG3 were linked to adaptive organization in late and severe rejection. IgM slightly increased early on, while IgE and C4d tended to increase in late severe rejection. Humoral activation was absent in isografts (**Supplementary Figure S10**).

## Discussion

This orthotopic lung transplant model, including a major MHC mismatch with immunosuppression, is the first to examine the nature, timing, and place of chronic rejection after lung transplantation. The methodological approach combining imaging, histology, and transcriptomic profiling allows the observation of chronic rejection from pathology to immunology.

We revealed the true nature of chronic rejection after lung transplantation, originating around vessels and, more precisely, around the arterioles. After innate activation, adaptive activation and fibrosis around arteries resulted in end-arteritis obliterans. Only later did innate venous inflammation, pleural infiltration/fibrosis, and “obliterative bronchiolitis” appear. μCT imaging confirmed that the gradual rejection model within 10 weeks was reproducible. The gradual onset of rejection questions the segregation of rejection into hyperacute, acute, and chronic rejection. Both cellular and humoral immunity may be part of the same immune response to rejection, where only the timing and magnitude differ.

The most important finding is the endothelial origin of chronic rejection, which alludes to abandoning the old enigma of airway-centered rejection, “obliterative bronchiolitis”. The concept that recipients’ immune cells only identify foreign cells in the small airway as “non-self” and induce the rather limited immune organization of the OB is counterintuitive. Although previous mouse lung transplant studies identified intraluminal airway fibrosis and constriction, including pleuro-parenchymal infiltration and fibrosis (10, 12, 20, 21), this study identified the first site of chronic rejection as being at the arteriole site. This new observation is in line with all solid organ transplantations (22) and is more plausible as recipients’ immune cells enter the “foreign” donor via arteries, representing the first contact location.

μCT imaging opened unique insights into the progression of rejection. Early innate onset and adaptive immune activation around the arteries and venous compartment are presented as mild lung attenuation. Severe attenuation is present when fibrotic organization around arteries, venous innate inflammation, and pleural and airway inflammation are histologically found.

Histological imaging of chronic rejection in both time and place was confirmed by single-cell RNA profiling. The earliest event of (chronic) rejection—endothelial activation—was observed by the upregulation of MHC1/2, adhesion molecules, and integrins, initiating extravasation of innate and adaptive immune cells. Within T helper cells, early master, inducer, and effector cytokines were not only increasingly expressed, demonstrating lymphoid activation, but also blocked regulation as Treg cells. Rejection is not only about immune cells controlling homeostasis but also about structural and stromal cells. Low expression levels in structural cells and stromal cells indicate that the lung structure is under pressure and its homeostasis is lost. Although adventitial fibroblasts appear to be the leading producers of ECM in rejection, multiple stromal cells were identified as ECM drivers, supporting the idea that rejection is more than restricted to OB lesions. In addition to the B cell involvement found by histology and cell profiling, humoral elements such as IgG2c and IgG3 confirmed the adaptive response in late rejection, in line with delayed-type hypersensitivity of rejection. Obviously, cell profiling should be more mechanistically validated.

This murine model, with its diagnostic tool, opens new horizons. This study maps (chronic) rejection and confirms its standard immune response nature, as we have only one immune system. Since all cells, cytokines, and so on resemble the classical immune responses, it is difficult to consider the specificity of rejection. Immune responses against microbial and malignant cells or immune responses due to secondary immune deficiency may have seriously biased our understanding of rejection. All can be studied in this controlled setting by paralleling infections, environmental factors, medication non-adherence, and autoimmune-induced immune responses. In addition, this model opens perspectives for immunotherapy research.

Limitations are the low *n*-values, the presence of failures, and the heterogeneity of rejection. To prevent failures, it is important to identify infections, twisted cuffs, and air leaks. The heterogeneity in the progression and severity of rejection may be related to surgical processes such as suturing difficulties, flushing issues, twisted cuffs, and the uptake of immunosuppression.

Our goal to validate the histological and imaging findings of rejection was achieved very elegantly and provided new avenues for research. Where chronic rejection fits into the clinical hallmarks of BOS and RAS is not clear-cut anymore, and how these mouse findings of rejection parallel the spectrum of CLAD remains to be answered. The observed lesions in the mouse are most consistent with RAS. Patients with BOS may experience early chronic rejection, but the pronounced airway pathology caused by immunosuppression or excessive exposure through inhalation of microorganisms and pollution may be confused with rejection. BOS and RAS are different but may have more overlap than identified.

This study described the true nature, timing, and location of chronic rejection after lung transplantation in murine orthotopic lung transplantation using cutting-edge diagnostic tools and opened new horizons for research. It invites researchers to re-explore chronic rejection in the clinical setting of CLAD.

## Data availability statement

The single cell RNA dataset presented in the study are deposited in the NCBI SRA bioproject, accession number PRJNA1076139: The nature of chronic rejection after lung transplantation: a murine orthotopic lung transplant study.

## Ethics statement

The animal study was approved by the Ethics Committee for Animal Research at KU Leuven. The study was conducted in accordance with local legislation and institutional requirements.

## Author contributions

TH: Data curation, Formal analysis, Investigation, Methodology, Writing – original draft. JK: Formal analysis, Investigation, Methodology, Writing – review & editing. CA: Formal analysis, Investigation, Methodology, Writing – review & editing. JSe: Investigation, Methodology, Writing – review & editing. YY: Formal analysis, Methodology, Writing – review & editing. VG: Methodology, Writing – review & editing, Investigation, Visualization. AH: Data curation, Formal analysis, Writing – review & editing, Investigation, Methodology. ArV: Investigation, Methodology, Writing – review & editing, Formal analysis. AS: Methodology, Writing – review & editing, Formal analysis, Investigation. SO: Methodology, Writing – review & editing, Formal analysis. AF: Investigation, Methodology, Formal analysis, Writing – review & editing. BS-G: Writing – review & editing, Data curation, Formal analysis, Visualization. JSl: Investigation, Methodology, Writing – review & editing. HB: Investigation, Methodology, Writing – review & editing, Data curation. NA: Methodology, Writing – review & editing, Visualization. MO: Methodology, Writing – review & editing, Investigation. AnV: Investigation, Methodology, Writing – review & editing. SC: Formal analysis, Methodology, Writing – review & editing, Software. DS: Methodology, Writing – review & editing, Formal analysis, Supervision, Validation. GV: Methodology, Writing – review & editing, Formal analysis, Supervision. JSc: Formal analysis, Methodology, Writing – review & editing, Data curation, Investigation. NK: Methodology, Supervision, Writing – review & editing, Funding acquisition, Investigation, Project administration. MB: Data curation, Formal analysis, Methodology, Writing – review & editing. HK: Funding acquisition, Supervision, Writing – review & editing, Resources. SM: Methodology, Writing – review & editing, Resources, Visualization. LD: Resources, Supervision, Writing – review & editing, Project administration. JV: Methodology, Writing – review & editing, Conceptualization, Supervision. LG: Supervision, Writing – review & editing, Data curation, Methodology. DR: Methodology, Conceptualization, Data curation, Investigation, Supervision, Writing – original draft. WJ: Supervision, Writing – review & editing, Funding acquisition, Project administration, Resources. GG-R: Funding acquisition, Project administration, Supervision, Writing – review & editing, Investigation, Methodology. LC: Investigation, Methodology, Supervision, Writing – review & editing. JM: Data curation, Methodology, Software, Writing – review & editing. EV:Methodology, Conceptualization, Data curation, Investigation, Supervision, Writing – original draft. RV: Conceptualization, Funding acquisition, Supervision, Writing – review & editing. BV: Conceptualization, Data curation, Formal analysis, Funding acquisition, Investigation, Methodology, Project administration, Resources, Supervision, Validation, Visualization, Writing – original draft, Writing – review & editing.

## Supplementary material

Supplementary Video 1 Representative macroscopy, including lung ventilation, microscopy, and µCT of the different pathological presentations. The different patterns include fully normal lungs, completely destroyed failures, and lungs demonstrating chronic rejection with a spectrum of extreme, severe, and mild rejection.

Supplementary Figure 2 Postoperative body weight and cyclosporine trough levels. The cyclosporine trough level of all mice was 587 ± 35µg/L, within the aimed range related to a better outcome for mice.

Supplementary Figure 4 Morphometric longitudinal *in vivo* µCT lung evolution of the native right lung for lung volume and parenchymal attenuation. At each time point, either individual values or the median with SEM are presented.

Supplementary Video 2 Representative longitudinal *in vivo* µCT lung reconstruction of the representative grafts. I2, I4, A20, and A22 are represented by inspiration and expiration scans, and three rotating and ventilating 3D reconstructions of the scans at 7, 35, and 70 days.

Supplementary Video 3 Representative *ex vivo* µCT lung reconstruction of an isograft and mild allografts. The airways (light blue), arterial (pink), and venous (red) systems of I9 and A28 (day 70) are segmented and reconstructed in 3D.

Supplementary Figure 6 Tidal volume of the different presentations of rejection. From the representative graft (I2, I4; A20 A22) the lung volume difference between in and expiration was calculated for each time point of the whole lung (upper panel), the right native lung (left side), and the left transplanted graft lung (right side). In the lower panel, a subdivision is made for PGD in the isograft group and for mild versus severe in the allograft group.

Supplementary Figure 7 Single-cell RNA profiling of the different cells of the structural cell subcluster. **(A)** UMAP plot of all cells of the left lung color-coded by major cellular lineage. **(B)** UMAP plot of the structural cells of the left lung, color-coded by the cellular subcluster of the structural cells. **(C)** Dot plot heatmap of the structural cell subcluster. The size and color intensity of each dot represent, respectively, the percentage of cells within each cell type. Color scale: red, high expression; blue, low expression. **(D)** A heatmap of the expression of mouse MHC1/2 complex H-2 genes within the endothelial cell lineages, where each line represents a specific endothelial cell and the color intensity represents the expression of the specific H2 gene. Color scale: yellow, high expression; purple, low expression. **(E)** Violin plots of proteasome elements, chemokines, and interferon pathway members were divided into the control mouse and isograft and allograft at days 7, 35, and 70. **(F)** UMAP plot of lung cells, color-coded for different adhesion molecules. Color scale: purple, high expression; grey, low expression. G**/**UMAP plot of lung cells for controls, isografts, and allografts, color-coded for the adhesion molecule CD34. Color scale: purple, high expression; grey, low expression.

Supplementary Figure 8 Single-cell RNA profiling of the different cells of the immune cell subcluster. **(A)** UMAP plot of all cells of the left lung, color-coded by major cellular subcluster. **(B)** UMAP plot of the immune cells of the left lung color-coded by the cellular subcluster of the immune cells. **(C)** Dot plot heatmap of the immune cell subcluster. The size and color intensity of each dot represent, respectively, the percentage of cells within each cell type. Color scale: red, high expression; blue, low expression. **(D)** Violin plots of T cell activation elements specific to the T cell subcluster divided into the control mouse, isograft, and allograft at days 7, 35, and 70. **(E)** and **(G)** Heatmaps of gene expression of genes correlated with pseudotime disease progression (Pseudotime: red to blue) within innate macrophages or adaptive T cells. Gene expression was scaled and plotted from low (purple) to high (yellow) expression.

Supplementary Figure 9 Single-cell RNA profiling of the different cells of the stromal cell subcluster. **(A)** UMAP plot of all cells of the left lung, color-coded by major cellular cluster. **(B)** UMAP plot of the structural cells of the left lung, color-coded by the cellular cluster of the stromal cells. **(C)** Dot plot heatmap of the stromal cell subcluster. The size and color intensity of each dot represent, respectively, the percentage of cells within each cell type. Color scale: red, high expression; blue, low expression. **(D)** Dot plot heatmap of expression of representative marker genes of contractility and extracellular matrix proteins for the different groups, including control, isograft, and allograft (with time points). The size and color intensity of each dot represent respectively the percentage or average expression of the marker gene in this cell type. Color scale: blew, high expression; white, low expression. **(E)** Connectome showing ligand-receptor pairs identified within control, isograft, or allograft mice. Colors denote the different cell types. **(F)** Connectome showing ligand-receptor pairs identified in the different allograft timepoints (A1W, A5W, or A10W). It should be noted that collagen ligands were highly connected in the early stages of rejection.

Supplementary Figure 10 Serial evaluation of systemic immunoglobulins and complement factors. Immunoglobulins included are IgG1, IgG2b, IgG2c, IgG3, IgM, IgA, and IgE accompanied by IgG against double strained DNA, BL6 DNA, and BalBc DNA. For complement, C4d was used.

## References

[1] Sayegh MH Carpenter CB. Transplantation 50 years later - progress, challenges, and promises. New Engl J Med. (2004) 351:2761–6. doi: 10.1056/NEJMon043418 15616214

[2] Li XC Jevnikar AM. Transplant immunology. Houston, USA: Wiley-Blackwell (2015). doi: 10.1002/9781119072997

[3] Verleden GM Glanville AR Lease ED Fisher AJ Calabrese F Corris PA. Chronic lung allograft dysfunction: Definition, diagnostic criteria, and approaches to treatment-A consensus report from the Pulmonary Council of the ISHLT. J Heart Lung Transplant. (2019) 38:493–503. doi: 10.1016/j.healun.2019.03.009 30962148

[4] Barker AF Bergeron A Rom WN Hertz MI. Obliterative bronchiolitis. N Engl J Med. (2014) 370:1820–8. doi: 10.1056/NEJMra1204664 24806161

[5] Sato M Waddell TK Wagnetz U Roberts HC Hwang DM Haroon A. Restrictive allograft syndrome (RAS): a novel form of chronic lung allograft dysfunction. J Heart Lung Transplant. (2011) 30:735–42. doi: 10.1016/j.healun.2011.01.712 21419659

[6] Okazaki M Krupnick AS Kornfeld CG Lai JM Ritter JH Richardson SB. A mouse model of orthotopic vascularized aerated lung transplantation. Am J Transplant. (2007) 7:1672–9. doi: 10.1111/j.1600-6143.2007.01819.x 17511692

[7] Jungraithmayr WM Korom S Hillinger S Weder W. A mouse model of orthotopic, single-lung transplantation. J Thorac Cardiovasc Surg. (2009) 137:486–91. doi: 10.1016/j.jtcvs.2008.10.007 19185174

[8] Suzuki H Fan L Wilkes DS. Development of obliterative bronchiolitis in a murine model of orthotopic lung transplantation. J Vis Exp. (2012) 65. doi: 10.3791/3947 PMC3476401 22805894

[9] Oishi H Martinu T Sato M Matsuda Y Hirayama S Juvet SC. Halofuginone treatment reduces interleukin-17A and ameliorates features of chronic lung allograft dysfunction in a mouse orthotopic lung transplant model. J Heart Lung Transplant. (2016) 35:518–27. doi: 10.1016/j.healun.2015.12.003 26787621

[10] Martinu T Oishi H Juvet SC Cypel M Liu M Berry GJ. Spectrum of chronic lung allograft pathology in a mouse minor-mismatched orthotopic lung transplant model. Am J Transplant. (2019) 19:247–58. doi: 10.1111/ajt.15167 30378739

[11] De Vleeschauwer S Jungraithmayr W Wauters S Willems S Rinaldi M Vaneylen A. Chronic rejection pathology after orthotopic lung transplantation in mice: the development of a murine BOS model and its drawbacks. PloS One. (2012) 7:e29802. doi: 10.1371/journal.pone.0029802 22238655 PMC3253086

[12] Yamada Y Vandermeulen E Heigl T Somers J Vaneylen A Verleden S. The role of recipient derived interleukin-17A in a murine orthotopic lung transplant model of restrictive chronic lung allograft dysfunction. Transplant Immunol. (2016) 39:10–7. doi: 10.1016/j.trim.2016.10.001 27737799

[13] Berghen N Dekoster K Marien E Dabin J Hillen A Wouters J. Radiosafe micro-computed tomography for longitudinal evaluation of murine disease models. Sci Rep. (2019) 9:17598. doi: 10.1038/s41598-019-53876-x 31772203 PMC6879529

[14] De Langhe E Vande Velde G Hostens J Himmelreich U Nemery B Luyten FP. Quantification of lung fibrosis and emphysema in mice using automated micro-computed tomography. PloS One. (2012) 7:e43123. doi: 10.1371/journal.pone.0043123 22912805 PMC3418271

[15] Vande Velde G Poelmans J De Langhe E Hillen A Vanoirbeek J Himmelreich U. Longitudinal micro-CT provides biomarkers of lung disease that can be used to assess the effect of therapy in preclinical mouse models and reveal compensatory changes in lung volume. Dis Models Mechanisms. (2016) 9 (1):91–8. doi: 10.1242/dmm.020321 PMC4728330 26563390

[16] Yushkevich PA Piven J Hazlett HC Smith RG Ho S Gee JC. User-guided 3D active contour segmentation of anatomical structures: Significantly improved efficiency and reliability. NeuroImage. (2006) 31:1116–28. doi: 10.1016/j.neuroimage.2006.01.015 16545965

[17] Adams TS Schupp JC Poli S Ayaub EA Neumark N Ahangari F. Single-cell RNA-seq reveals ectopic and aberrant lung-resident cell populations in idiopathic pulmonary fibrosis. Sci Adv. (2020) 6:eaba1983. doi: 10.1126/sciadv.aba1983 32832599 PMC7439502

[18] Yu G Wang L-G Han Y He Q-Y. clusterProfiler: an R package for comparing biological themes among gene clusters. OMICS. (2012) 16:284–7. doi: 10.1089/omi.2011.0118 PMC3339379 22455463

[19] Supek F Bošnjak M Škunca N Šmuc T. REVIGO summarizes and visualizes long lists of gene ontology terms. PloS One. (2011) 6:e21800. doi: 10.1371/journal.pone.0021800 21789182 PMC3138752

[20] Fan L Benson HL Vittal R Mickler EA Presson R Jo Fisher A. Neutralizing IL-17 prevents obliterative bronchiolitis in murine orthotopic lung transplantation. Am J Transplant. (2011) 11:911–22. doi: 10.1111/j.1600-6143.2011.03482.x PMC3083638 21521466

[21] Misumi K Wheeler DS Aoki Y Combs MP Braeuer RR Higashikubo R. Humoral immune responses mediate the development of a restrictive phenotype of chronic lung allograft dysfunction. JCI Insight. (2020) 5. doi: 10.1172/jci.insight.136533 PMC7714414 33268593

[22] Libby P Pober JS. Chronic rejection. Immunity. (2001) 14:387–97. doi: 10.1016/S1074-7613(01)00119-4 11336684

