# Supplementary figures and images for: The nature of chronic rejection after lung transplantation: a murine orthotopic lung transplant study
Source: Front Immunol. 2024 Apr 25;15:1369536. doi: 10.3389/fimmu.2024.1369536 (PMC11084670; doi:10.3389/fimmu.2024.1369536)

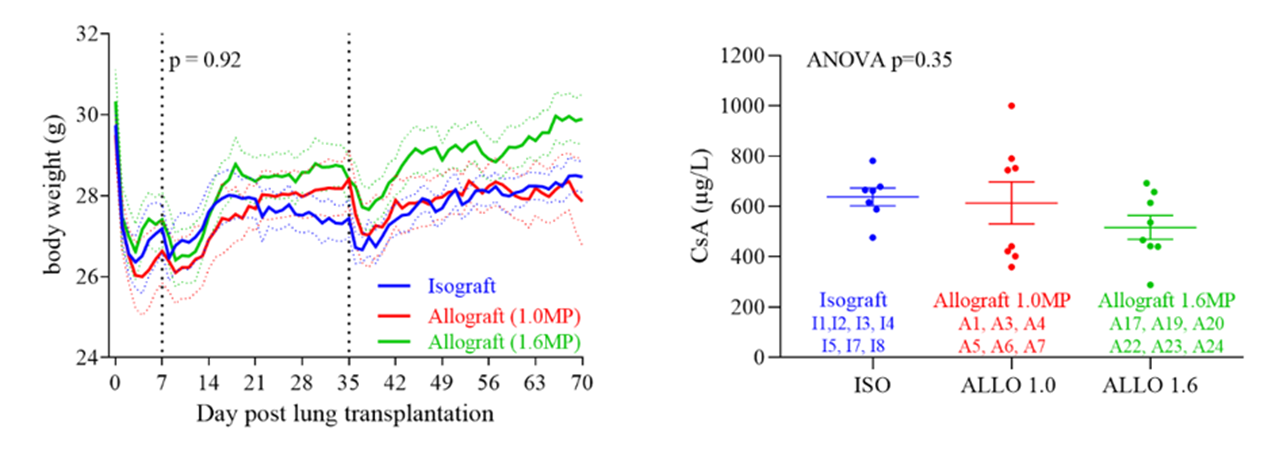

Supplement: Supplementary Video 2 — Representative longitudinal in vivo µCT lung reconstruction of the representative grafts. I2, I4, A20, and A22 are represented by inspiration and expiration scans, and three rotating and ventilating 3D reconstructions of the scans at 7, 35, and 70 days. [file Image_2.png]

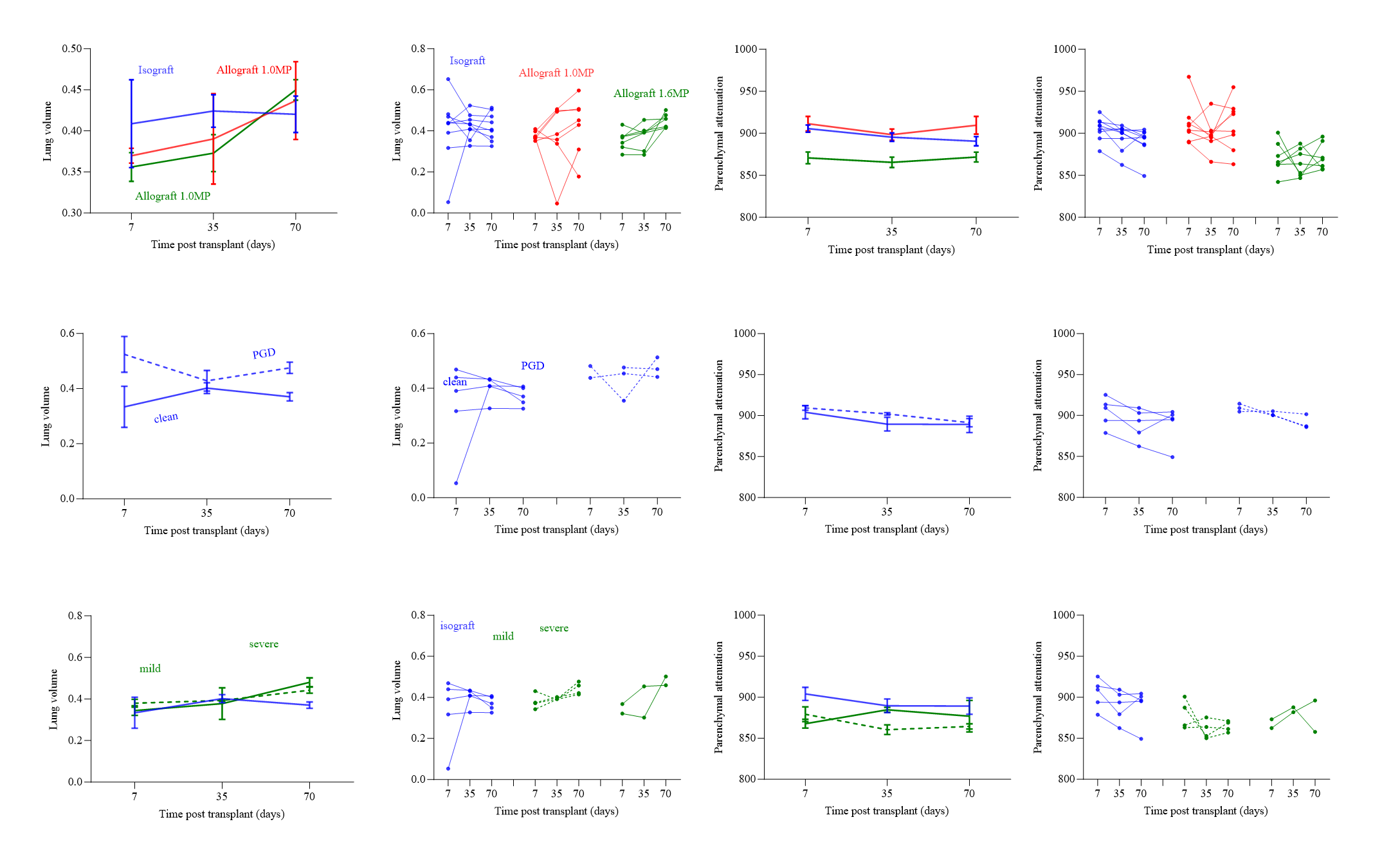

Supplement: Supplementary Video 3 — Representative ex vivo µCT lung reconstruction of an isograft and mild allografts. The airways (light blue), arterial (pink), and venous (red) systems of I9 and A28 (day 70) are segmented and reconstructed in 3D. [file Image_4.tif]

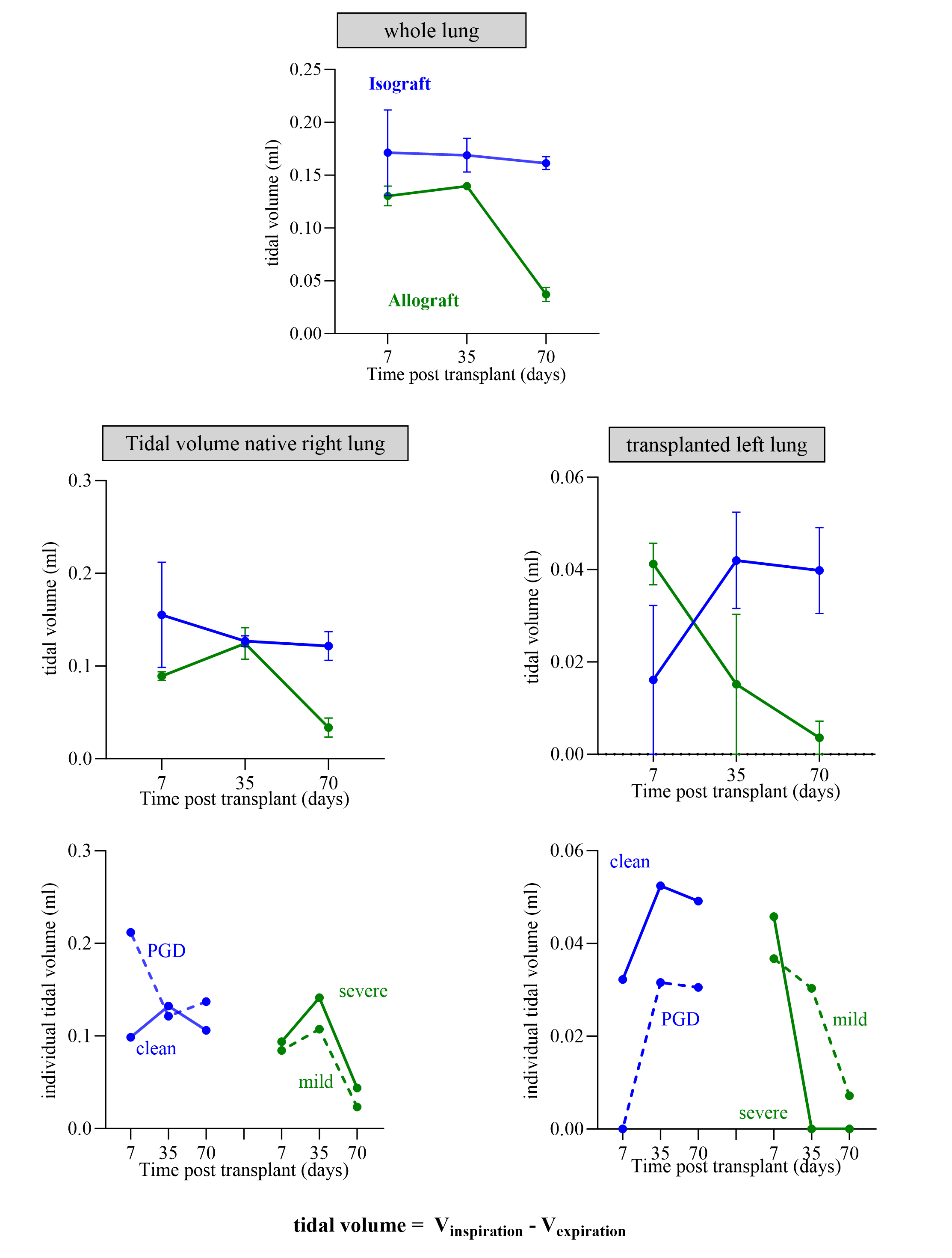

Supplement: Supplementary Figure 6 — Tidal volume of the different presentations of rejection. From the representative graft (I2, I4; A20 A22) the lung volume difference between in and expiration was calculated for each time point of the whole lung (upper panel), the right native lung (left side), and the left transplanted graft lung (right side). In the lower panel, a subdivision is made for PGD in the isograft group and for mild versus severe in the allograft group. [file Image_6.tif]

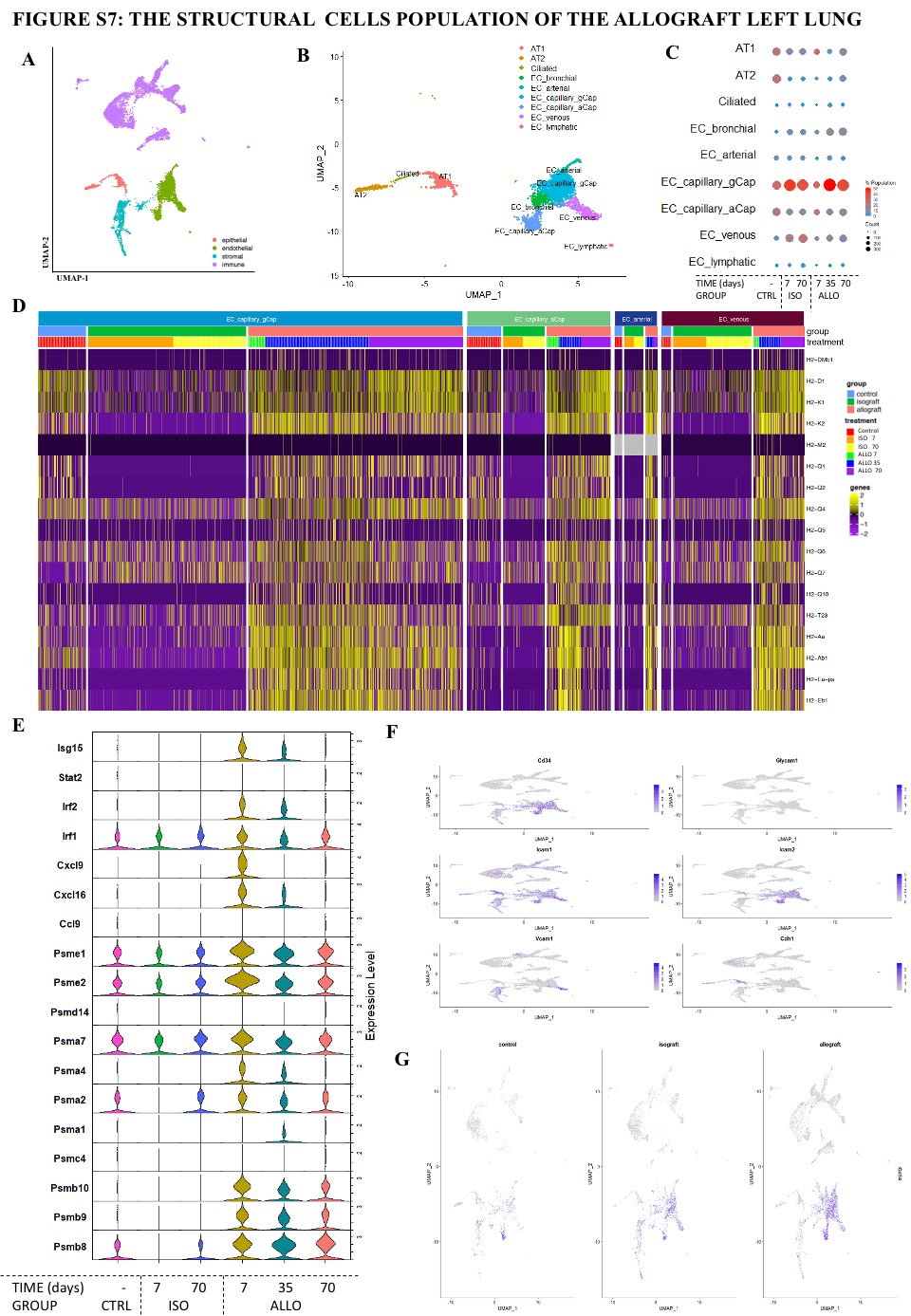

Supplement: Supplementary Figure 7 — Single-cell RNA profiling of the different cells of the structural cell subcluster. (A) UMAP plot of all cells of the left lung color-coded by major cellular lineage. (B) UMAP plot of the structural cells of the left lung, color-coded by the cellular subcluster of the structural cells. (C) Dot plot heatmap of the structural cell subcluster. The size and color intensity of each dot represent, respectively, the percentage of cells within each cell type. Color scale: red, high expression; blue, low expression. (D) A heatmap of the expression of mouse MHC1/2 complex H-2 genes within the endothelial cell lineages, where each line represents a specific endothelial cell and the color intensity represents the expression of the specific H2 gene. Color scale: yellow, high expression; purple, low expression. (E) Violin plots of proteasome elements, chemokines, and interferon pathway members were divided into the control mouse and isograft and allograft at days 7, 35, and 70. (F) UMAP plot of lung cells, color-coded for different adhesion molecules. Color scale: purple, high expression; grey, low expression. G/UMAP plot of lung cells for controls, isografts, and allografts, color-coded for the adhesion molecule CD34. Color scale: purple, high expression; grey, low expression. [file Image_7.jpeg]

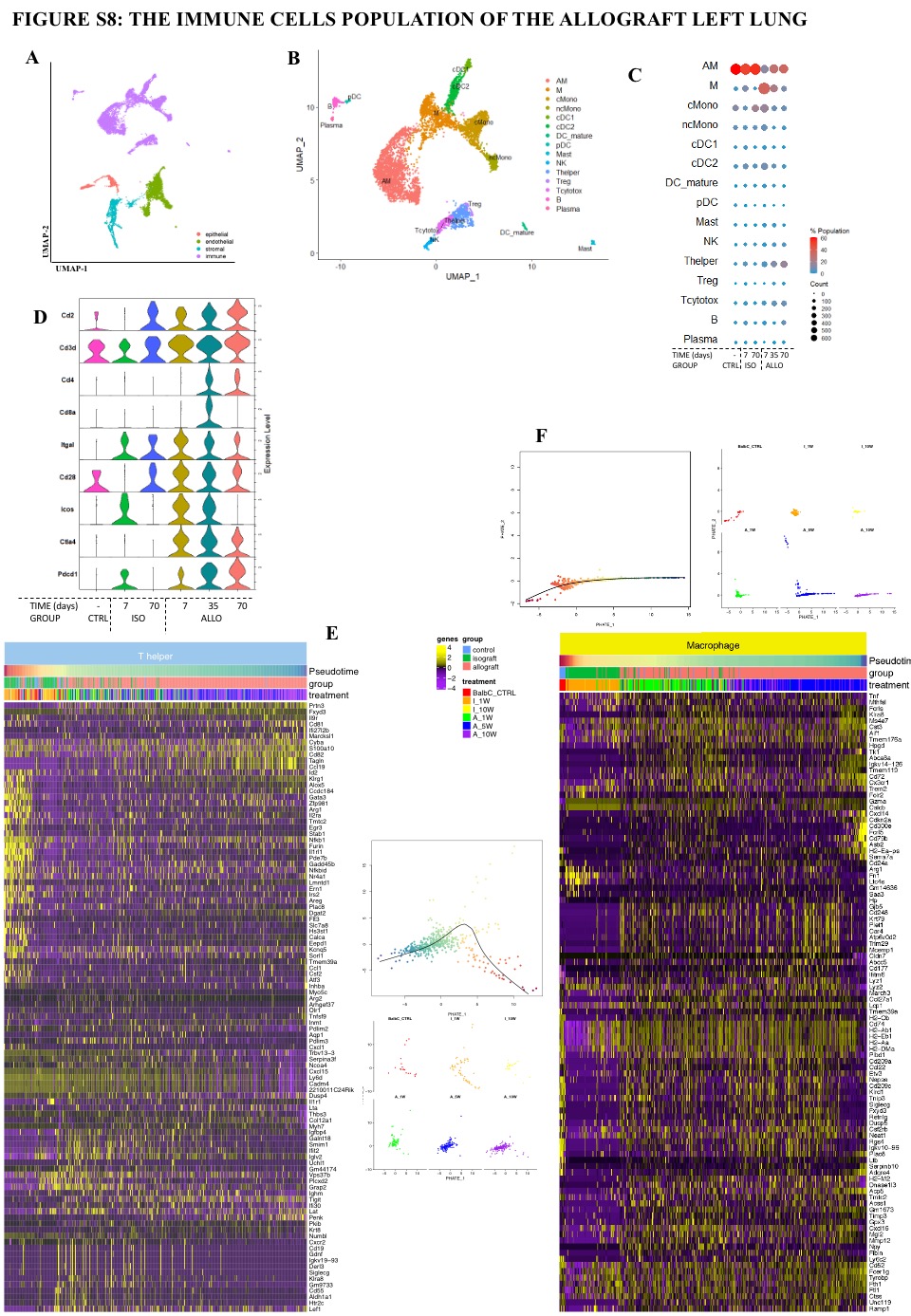

Supplement: Supplementary Figure 8 — Single-cell RNA profiling of the different cells of the immune cell subcluster. (A) UMAP plot of all cells of the left lung, color-coded by major cellular subcluster. (B) UMAP plot of the immune cells of the left lung color-coded by the cellular subcluster of the immune cells. (C) Dot plot heatmap of the immune cell subcluster. The size and color intensity of each dot represent, respectively, the percentage of cells within each cell type. Color scale: red, high expression; blue, low expression. (D) Violin plots of T cell activation elements specific to the T cell subcluster divided into the control mouse, isograft, and allograft at days 7, 35, and 70. (E) and (G) Heatmaps of gene expression of genes correlated with pseudotime disease progression (Pseudotime: red to blue) within innate macrophages or adaptive T cells. Gene expression was scaled and plotted from low (purple) to high (yellow) expression. [file Image_8.jpeg]

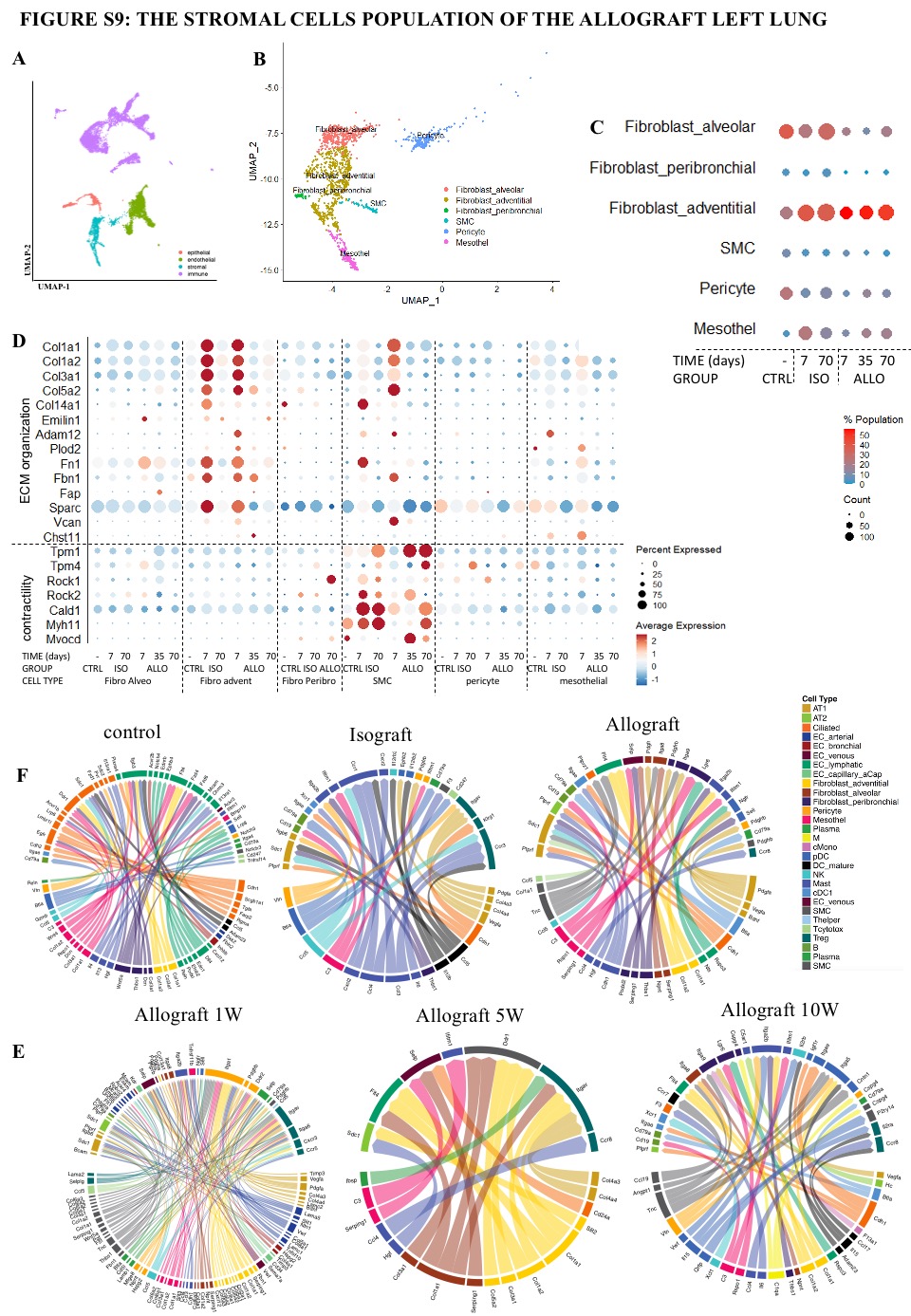

Supplement: Supplementary Figure 9 — Single-cell RNA profiling of the different cells of the stromal cell subcluster. (A) UMAP plot of all cells of the left lung, color-coded by major cellular cluster. (B) UMAP plot of the structural cells of the left lung, color-coded by the cellular cluster of the stromal cells. (C) Dot plot heatmap of the stromal cell subcluster. The size and color intensity of each dot represent, respectively, the percentage of cells within each cell type. Color scale: red, high expression; blue, low expression. (D) Dot plot heatmap of expression of representative marker genes of contractility and extracellular matrix proteins for the different groups, including control, isograft, and allograft (with time points). The size and color intensity of each dot represent respectively the percentage or average expression of the marker gene in this cell type. Color scale: blew, high expression; white, low expression. (E) Connectome showing ligand-receptor pairs identified within control, isograft, or allograft mice. Colors denote the different cell types. (F) Connectome showing ligand-receptor pairs identified in the different allograft timepoints (A1W, A5W, or A10W). It should be noted that collagen ligands were highly connected in the early stages of rejection. [file Image_9.jpeg]

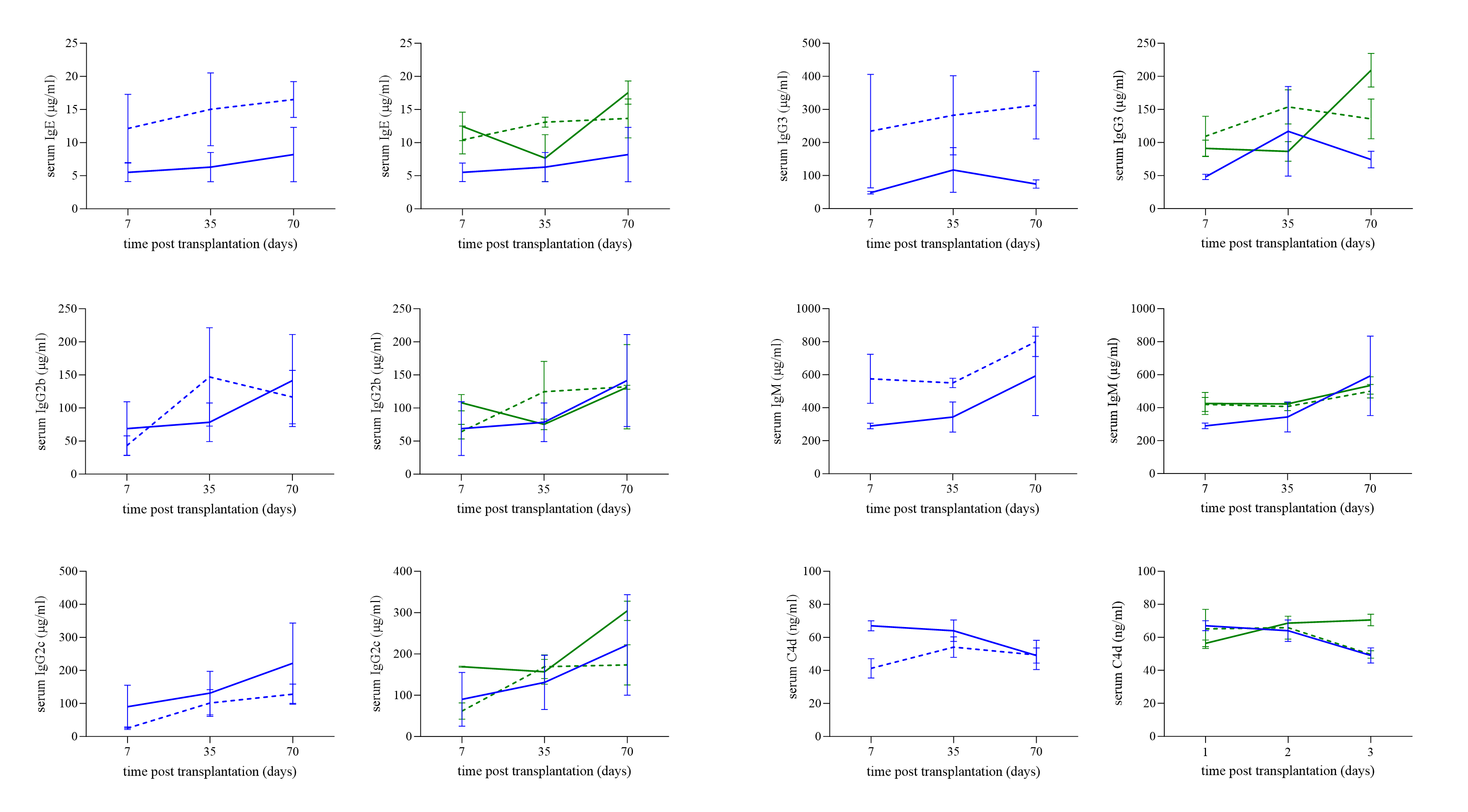

Supplement: Supplementary Figure 10 — Serial evaluation of systemic immunoglobulins and complement factors. Immunoglobulins included are IgG1, IgG2b, IgG2c, IgG3, IgM, IgA, and IgE accompanied by IgG against double strained DNA, BL6 DNA, and BalBc DNA. For complement, C4d was used. [file Image_10.tif]
